# Comparison between thoracic low-dose computed tomography and conventional-dose computed tomography in evaluating anemia: A preliminary study in a Chinese screening cohort

**DOI:** 10.3389/fcvm.2022.987753

**Published:** 2022-10-26

**Authors:** Jianing Zhang, Minghao Wu, Jinchao Huang, Shixia Li, Zhaoxiang Ye

**Affiliations:** ^1^Department of Radiology, Tianjin Medical University Cancer Institute and Hospital, National Clinical Research Center for Cancer, Key Laboratory of Cancer Prevention and Therapy, Tianjin’s Clinical Research Center for Cancer, Tianjin, China; ^2^Department of Radiology, Beijing Tiantan Hospital, Capital Medical University, Beijing, China; ^3^Department of Cancer Prevention, Tianjin Medical University Cancer Institute and Hospital, Tianjin, China

**Keywords:** anemia, low-dose computed tomography, left ventricular cavity, descending aorta, hemoglobin

## Abstract

**Purpose:**

To investigate and evaluate the value of thoracic low dose computed tomography (LDCT) scan in the diagnosis of anemia.

**Materials and methods:**

661 patients who received thoracic computed tomography (CT) examination and underwent a peripheral blood examination were retrospectively included. 341 patients underwent conventional dose CT (CDCT), and 320 patients underwent LDCT. Regions of interest (ROI) were placed on the left ventricular cavity (LV), descending aorta (DAo), and interventricular septum (IVS). The corresponding CT attenuation was measured, and the CT attenuation difference between LV and IVS (IVS-LV) and between DAo and IVS (IVS-DAo) was calculated, respectively. One-way analysis of variance (ANOVA) and linear regression were performed to analyze the relationship between these indicators and Hb levels. The receiver operating characteristic (ROC) curve was used to evaluate prediction performance.

**Results:**

Both attenuation on LDCT and CDCT showed significant differences between the healthy group and the anemic group (*P* < 0.05). In the LDCT group, the LV and DAo were more relevant with the hemoglobin (Hb) level (correlation coefficient 0.618 and 0.602) than other indicators, with AUCs of 0.815 (95% CI: 0.763–0.868) and 0.803 (95% CI: 0.747–0.859), respectively. The linear regression formulas for Hb level with the LV and DAo were 19.14 + 0.15 × HU [95% CI: (16.52, 21.75) + (0.12, 0.17) × HU] and 19.46 + 0.16 × HU [95% CI: (16.55, 22.36) + (0.13, 0.18) × HU], respectively. Youden’s index indicated that 37.5 HU and 38.5 HU were the best thresholds to diagnose anemia for LV and DAo, respectively. In the CDCT group, the LV and IVS-LV got obviously higher correlation coefficients (0.813 and 0.812), with AUCs of 0.831 (95% CI: 0.786–0.877) and 0.851 (95% CI: 0.808–0.894), respectively. The optimal thresholds for LV and IVS-LV were 40.5 HU and 9.5 HU, respectively.

**Conclusion:**

In LDCT examinations, an approximation of Hb level and detecting of anemia can be conducted based on simple attenuation measurements.

## Introduction

Low-dose CT (LDCT) screening was a key component of the screening programs for early detection and early treatment of lung cancer (1–3). There was a significant reduction with LDCT screening in the rates of deaths from both lung cancer and any cause, which confirm the superiority of LDCT screening (4–7). However, the high false-positive result rate may lead to overdiagnosis and is generally the cost driver of LDCT lung cancer screening (8–10). Thus, it is of significance to broaden the clinical use of LDCT screening. Some previous studies have explored the application of LDCT in assessing the risk of cardiovascular disease (CVD) through the detection of coronary artery calcification (CAC) and identifying osteoporosis by bone mineral density (BMD) measurement (11, 12). The non-invasive, low-radiation CT scan can obtain more additional clinical information and has a broad application prospect in health management.

According to the WHO, the incidence of anemia has reached a global number of up to 2.36 billion (13, 14). Many previous studies suggested that anemia may partly result in impaired physical performance (15), decreased health-related quality of life (16), and increased mortality (17–19). Computed tomography (CT) is an essential diagnostic method in a variety of clinical settings and diseases. Recent studies have shown that conventional-dose CT (CDCT) values of the blood pool (e.g., left ventricular cavity) are correlated with Hb level, which is useful for the objective diagnosis of anemia (20–27). However, due to the decrease of tube voltage or tube current in LDCT screening, the indicators of CDCT should not be used directly before experimental verification.

Based on the above factors, we hypothesized that LDCT can provide detection of anemia. In this study, we evaluated the correlation of each indicator and Hb level to clarify if LDCT can provide an opportunistic approach to indicating anemia, meanwhile maximizing the effectiveness of LDCT screening and improving extra health benefits of the participant.

## Materials and methods

### Patients

**FIGURE 1:**
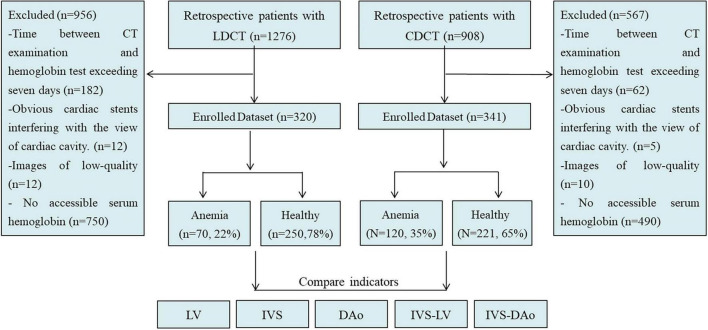
Patient flow diagram. LDCT, low dose computed tomography; CDCT, conventional dose computed tomography; IVS, interventricular septum; LV, left ventricular cavity; IVS-LV, CT attenuation difference between LV and IVS; DAo, descending aorta; IVS-DAo, CT attenuation difference between DAo and IVS.

The Medical Ethics Committee of Tianjin Medical University Cancer Hospital approved this retrospective study (No. Bc2018039) with a waiver of informed consent. From January 2019 to March 2019, we enrolled 908 patients who underwent CDCT and 1,276 patients who underwent LDCT according to the inclusion criteria (Figure 1): 1. Patients who received thoracic CDCT or LDCT; 2. Accessible serum Hb level. A total of 567 patients who underwent CDCT and 956 patients who underwent LDCT were excluded according to the exclusion criteria (Figure 1): 1. The time between CT examination and hemoglobin test exceeding 7 days; 2. Images with obvious motion artifact; 3. Obvious cardiac stents interfering with the view of the cardiac cavity. Finally, 341 patients who underwent CDCT and 320 patients who underwent LDCT were enrolled in the study. One of the patients who underwent an LDCT scan had a CDCT examination after finding lung abnormalities.

### Image acquisition and quality analysis

The subjects were randomly assigned to two equipment (SIEMENS SOMATOM Definition AS +, SIEMENS; GE MEDICAL SYSTEMS Discovery CT750 HD, GE) for CT scanning (181 by SIEMENS and 139 by GE in the LDCT group, and 156 by SIEMENS and 185 by GE in CDCT group, respectively). The reconstruction thickness of SIEMENS was 1.0 mm and that of GE equipment was 1.25 mm.

The subjective assessment of image quality was performed by two radiologists under the preset standard mediastinal settings (window width, 400 HU; window level, 60 HU). The two radiologists scored the image quality of the heart and aorta, respectively. Scoring criteria: ➀ 1 point: no noise, and clear structure; ➁ 2 points: slight noise, acceptable slight structure blur; ➂ 3 points: moderate noise and structure blur; ➃ 4 points: the noise is obvious and difficult to diagnose. The average score represents the image quality, 1–3 indicates that the diagnosis can be satisfied, and 4 indicates unable to diagnose. No case cannot be diagnosed. The consistency of the two radiologists’scores was good. The CT values and SD of the IVS, LV, and DAo lumen were recorded, and the contrast to noise ratio (CNR) was calculated to evaluate the image quality objectively. CNR = (SI_IVS_ –SI_lumen_)/noise. The noise was defined as the mean of the SD of the ROI of these measurements. There was no statistically significant difference in CNR between the two scanners.

**FIGURE 2:**
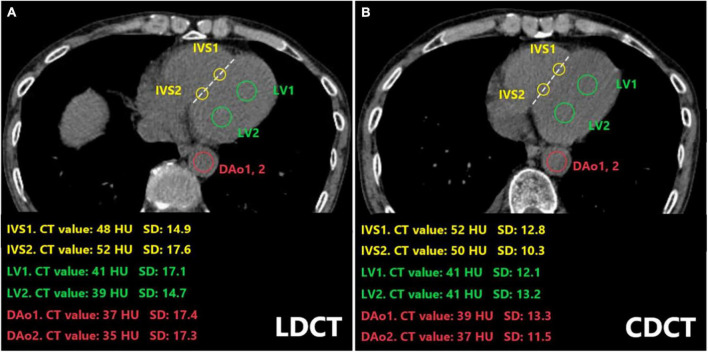
Placement of ROIs in the LV, IVS, and the DAo on CT images of a 64-year-old man who underwent both LDCT **(A)** and CDCT examination **(B).** The CT value and standard deviation for each ROI were indicated. ROIs on IVS were drawn along the line between the anterior interventricular sulcus and the anteriormost aspect of the left AV groove at the level of the junction of the coronary sinus and right atrium (dotted line). LDCT, low dose computed tomography; CDCT, conventional dose computed tomography; IVS, interventricular septum; LV, left ventricular cavity; DAo, descending aorta; HU, Hounsfield Unit; SD, standard deviation; ROI, Regions of interest.

For the conventional-dose scanning protocol, the tube voltage is 120 kV, and the tube current is set with automatic control of exposure. For the low-dose scanning protocol, the tube voltage is 120 kV, and the tube current is set with 35 mAs. All patients underwent a plain CT scan from the thoracic inlet to the first lumbar vertebra in a supine position. Two ROIs were selected from each LV, IVS, and DAo. For each ROI, CT value and standard deviation (SD) were recorded and averaged results of two ROIs were used for statistical analysis. Meanwhile, the differences in CT attenuation between LV and IVS (IVS-LV) and between DAo and IVS (IVS-DAo) were calculated, respectively. All parameters were measured independently by 2 radiologists who were blind to each patient’s information. To determine the measurement consistency within the observer, the measurement was, respectively, repeated by radiologists a week later. Referring to the previous study (25) and the echocardiographic measurements in normal Chinese adults (28), ROIs for the LV were taken at the size of 100 mm^2^ to avoid capturing the left ventricular wall and papillary muscle (mean LV internal diameter: 46.2 ± 4.0 mm for males and 43.2 ± 3.3 mm for females, respectively). To avoid the ROI diameter greater than the thickness of IVS (mean IVS thickness: < 8.9 mm for males and < 8.1 mm for females, respectively), the sizes of ROIs were maintained at less than 50 mm^2^. Draw a line between the anterior interventricular sulcus and the posterior interventricular sulcus while difficulty was encountered during delineation of the IVS. The diameter of ROI selected in the DAo was controlled at less than 15 mm to avoid vascular walls and artifacts from the spine as much as possible (Figure 2).

### Serum hemoglobin

A recent laboratory test with Hb concentration was within 7 days before or after the CT examination. All patients were divided into two groups according to Chinese criteria of diagnostic anemia. Male patients with Hb ≥ 120 g/L and female patients with Hb ≥ 110 g/L were considered healthy. Male patients with Hb < 120 g/L and female patients with Hb < 110 g/L were assigned to the anemia group.

### Statistical analysis

One-way analysis of variance (ANOVA) and linear regression analysis were performed to analyze the relationship between these indicators and Hb levels. The least significant difference (LSD) multiple comparison method was used to compare differences among variables. A receiver operating characteristic (ROC) curve was used to evaluate the prediction performance of each indicator. In addition, to determine the optimal threshold CT value for diagnosing anemia, the sensitivity, specificity, and Youden’s index under the highest area under the curve (AUC) were calculated. Delong test was used to compare the differences between the detection efficiency of each index. All statistical analyses were performed with SPSS software version 22.0 (International Business Machines Corporation, Chicago Illinois, United States).

## Results

Intraclass correlation coefficient (ICC) was used to evaluate the consistency of measurements. The intra-observer ICC values of 2 radiologists and the inter-observer ICC value between 2 radiologists are 0.905, 0.901, and 0.810, respectively, indicating high consistency and repeatability of the measurements obtained (Table 1, *P* < 0.001). The consistency of subjective evaluation of image quality by two radiologists was evaluated by Kappa index (Table 2). The mean Kappa index of two radiologists was 0.82. There is no significant difference in image quality between the two groups.

**TABLE 1:** Comparison of intra-observer and inter-observer consistency and repeatability.

	ICC	95% CI
Intra-observer 1	0.905	0.858–0.937
Intra-observer 2	0.901	0.852–0.934
Inter-observer	0.810	0.752–0.856

ICC, intraclass correlation coefficient; 95% CI, 95% confidence interval.

**TABLE 2:** Subjective image quality scores of two radiologists.

	Radiologist A				Radiologist B				Kappa
			
	1	2	3	4	1	2	3	4	
**LDCT group**									
SIEMENS	149	27	5	0	158	23	0	0	0.75
GE	115	20	4	0	121	15	3	0	0.84
**CDCT group**									
SIEMENS	136	19	1	0	135	20	1	0	0.89
GE	166	18	1	0	166	19	0	0	0.80

LDCT, low dose computed tomography; CDCT, conventional dose computed tomography.

A total of 661 patients, 260 men and 401 women with a mean age of 58.20 ± 10.84 years (range 40–88 years) were enrolled in this study. Laboratory assessment of Hb levels ranged from 49 to 176 g/L (mean, 123.76 ± 21.17 g/L). The mean Hb level of the male was higher than that of the female (133.53 ± 22.33 g/L vs. 117.42 ± 17.73 g/L). Of these 661 patients, 320 patients underwent LDCT and 341 patients underwent CDCT. 337 scanned by SIEMENS, and 324 scanned by GE, respectively. No significant difference in CT values was observed on images of different scanners. Tables 3, 4 showed the detailed characteristics of Hb level and CT values of each indicator. In the LDCT group, the differences of each indicator between the healthy group and the anemia group were statistically significant (*P* < 0.05), and the differences of each indicator between different genders were statistically significant (*P* < 0.05) except for the IVS-LV (*P* = 0.103). In the CDCT group, the differences of each indicator between the healthy group and anemia group were statistically significant except for the IVS (*P* = 0.058), and the differences of each indicator between different genders were statistically significant except the IVS-DAo (*P* = 0.319).

**TABLE 3:** Descriptive analysis of baseline characteristics in CDCT group.

	Total (341)	Hb level			Gender		
			
		Anemia group (120)	Healthy group (221)	*P*	Male (167)	Female (174)	*P*
Age	56 (49.50–63)	60 (50–66.75)	55 (49–61)	0.006*	56 (49–64)	55 (50–62)	0.282
Hb (g/L)	120.82 ± 20.70	98.91 ± 13.28	132.72 ± 12.76	0.000*	126.30 ± 21.49	115.56 ± 18.50	0.000**
IVS (HU)	50 (48–51)	49 (48–50)	50 (48–51.5)	0.058	50 (48–52)	49 (47–50)	0.000**
LV (HU)	41 (37–44)	36 (32–40)	42 (39–45)	0.000*	42 (38–45)	39 (36–43)	0.000**
IVS-LV (HU)	9 (6–12)	12 (10–16)	7 (5–9)	0.000*	8 (5–12)	9 (7–12)	0.001**
DAo (HU)	40 (36–43)	36 (32.25–39)	42 (39–44)	0.000*	41 (37–44)	39 (35–42)	0.003**
IVS-DAo (HU)	10 (6–13)	13 (10–16)	8 (5–11)	0.000*	10 (6–13)	10 (7–13)	0.319

CDCT, conventional dose computed tomography; Hb, hemoglobin; IVS, interventricular septum; LV, left ventricular cavity; IVS-LV, CT attenuation difference between LV and IVS; DAo, descending aorta; IVS-DAo, CT attenuation difference between DAo and IVS; HU, Hounsfield Unit. *The difference is significant between the anemia group and the healthy group. **The difference is significant between males and females.

**TABLE 4:** Descriptive analysis of baseline characteristics in LDCT group.

	Total (320)	Hb level			Gender		
			
		Anemia group (70)	Healthy group (250)	*P*	Male(93)	Female (227)	*P*
Age	62 (40–87)	47 (40–50)	63 (40–87)	0.000*	65 (54–87)	55 (40–54)	0.000**
Hb (g/L)	126.89 ± 21.26	97.91 ± 11.42	135.00 ± 15.51	0.000*	146.53 ± 17.46	118.84 ± 17.03	0.000**
IVS (HU)	50 (37–67)	48 (39–60)	51 (37–67)	0.000*	52 (38–67)	49 (37–66)	0.000**
LV (HU)	38 (22–51)	33 (22–41)	40 (26–51)	0.000*	40 (26–51)	37 (22–46)	0.000**
IVS-LV (HU)	13 (2–29)	15 (6–28)	12 (2–29)	0.000*	12 (2–24)	13 (5–29)	0.103
DAo (HU)	40 (24–57)	35 (26–45)	40 (24–57)	0.000*	42 (26–57)	38 (24–51)	0.000**
IVS-DAo (HU)	11 (1–28)	13 (2–27)	11 (1–28)	0.002*	10 (1–28)	12 (1–27)	0.005**

LDCT, low dose computed tomography; Hb, hemoglobin; IVS, interventricular septum; LV, left ventricular cavity; IVS-LV, CT attenuation difference between LV and IVS; DAo, descending aorta; IVS-DAo, CT attenuation difference between DAo and IVS; HU, Hounsfield Unit. *The difference is significant between the anemia group and the healthy group. **The difference is significant between males and females.

**FIGURE 3:**
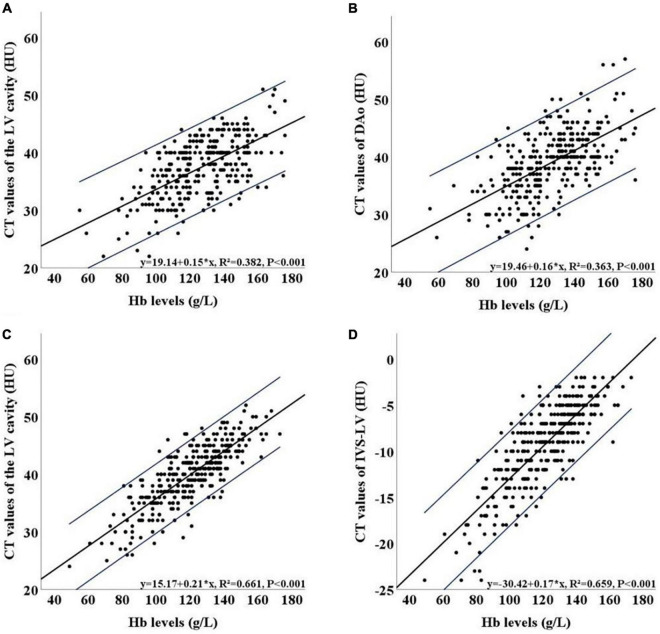
Scatter plots of the relationship between Hb level and the LV **(A)** and between Hb level and DAo **(B)** in the LDCT group. Scatter plots of the relationship between Hb level and the LV **(C)** and between Hb level and IVS-LV **(D)** in the CDCT group. 95% CI of the estimation equation is shown indicated in blue lines. LDCT, low dose computed tomography; CDCT, conventional dose computed tomography; LV, left ventricular cavity; IVS-LV, CT attenuation difference between LV and IVS; DAo, descending aorta; HU, Hounsfield Unit; Hb, hemoglobin; 95% CI, 95% confidence interval.

Correlation coefficients between all indicators and Hb levels are summarized in Tables 5, 6. In the LDCT group, the LV and DAo were more relevant with the Hb level (correlation coefficient: 0.618 and 0.602, respectively) than other indicators. The scatter plots indicated the relationship between Hb level and above two indicators with the linear regression formulas of *y* = 19.14 + 0.15x [95% CI: (16.52, 21.75) + (0.12, 0.17)x] and y = 19.46 + 0.16x [95% CI: (16.55, 22.36) + (0.13, 0.18)x] (Figures 3A,B), respectively. The correlation between IVS and Hb level (r = 0.332) was lower than that between blood pools and the Hb level. The IVS-LV and IVS-DAo showed a weak positive linear correlation with the Hb level (correlation coefficient 0.274 and 0.255, respectively). In the CDCT group, the LV and IVS-LV got higher correlation coefficients with Hb level (*r* = 0.813 and 0.812, respectively) than other indicators and showed an obvious linear distribution in the scatter plots (Figures 3C,D). According to the linear relationship, the linear regression formulas of two indicators with Hb level were y = 15.17 + 0.21x [95% CI: (13.24, 17.10) + (0.19, 0.22)x] and y = –30.42 + 0.17x [95% CI: (–32.06, –28.78) + (0.16, 0.19)x], respectively. The correlation coefficients of DAo and IVS-DAo with Hb level were 0.641 and 0.557, respectively. The IVS got the lowest correlation coefficient (*r* = 0.258). Except for the IVS, the correlation coefficients between the Hb level and all indicators in the CDCT group were higher than that in the LDCT group.

**TABLE 5:** Correlation coefficients of indicators with Hb level in CDCT group.

	IVS	*P*	LV	*P*	IVS-LV	*P*	DAo	*P*	IVS-DAo	*P*
Total	0.258*	0.000	0.813*	0.000	0.812*	0.000	0.641*	0.000	0.557*	0.000
**Gender**										
Male	0.251*	0.001	0.824*	0.000	0.832*	0.000	0.669*	0.000	0.609*	0.000
Female	0.170	0.025	0.774*	0.000	0.785*	0.000	0.588*	0.000	0.525*	0.000
**Hb level**										
Healthy group	0.343*	0.000	0.716*	0.000	0.650*	0.000	0.484*	0.000	0.309*	0.000
Anemic group	0.129	0.160	0.743*	0.000	0.766*	0.000	0.433*	0.000	0.379*	0.000

*Correlation is significant at the 0.01 level (2-tailed). CDCT, conventional dose computed tomography; Hb, hemoglobin; IVS, interventricular septum; LV, left ventricular cavity; IVS-LV, CT attenuation difference between LV and IVS; DAo, descending aorta; IVS-DAo, CT attenuation difference between DAo and IVS; HU, Hounsfield Unit.

**TABLE 6:** Correlation coefficients of indicators with Hb level in LDCT group.

	IVS	*P*	LV	*P*	IVS-LV	*P*	DAo	*P*	IVS-DAo	*P*
Total	0.332*	0.000	0.618*	0.000	0.274*	0.000	0.602*	0.000	0.255*	0.000
**Gender**										
Male	0.192	0.065	0.504*	0.000	0.286*	0.005	0.457*	0.000	0.228	0.028
Female	0.307*	0.000	0.592*	0.000	0.254*	0.000	0.544*	0.000	0.198*	0.003
**Hb level**										
Healthy group	0.259*	0.000	0.442*	0.000	0.103	0.104	0.498*	0.000	0.180*	0.004
Anemic group	0.062	0.612	0.532*	0.000	0.572*	0.000	0.284	0.017	0.280	0.019

*Correlation is significant at the 0.01 level (2-tailed). LDCT, low dose computed tomography; Hb, hemoglobin; IVS, interventricular septum; LV, left ventricular cavity; IVS-LV, CT attenuation difference between LV and IVS; DAo, descending aorta; IVS-DAo, CT attenuation difference between DAo and IVS; HU, Hounsfield Unit.

**FIGURE 4:**
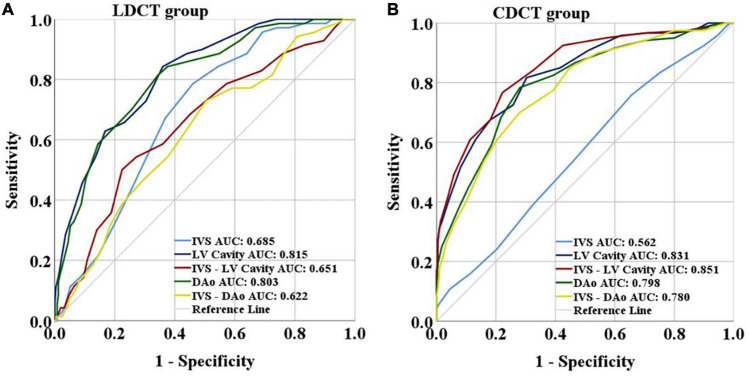
**(A)** Results of the ROC curve of LDCT group. **(B)** Results of the ROC curve of CDCT group. LDCT, low dose computed tomography; CDCT, conventional dose computed tomography; IVS, interventricular septum; LV, left ventricular cavity; IVS-LV, CT attenuation difference between LV and IVS; DAo, descending aorta; IVS-DAo, CT attenuation difference between DAo and IVS; ROC, receiver operating characteristic; AUC, area under the curve.

The results of the ROC curve are shown in Figures 4A,B. The best diagnostic indicators for anemia differed between the two groups. In the LDCT group, the LV and DAo had satisfactory performance in distinguishing anemia from non-anemia, with AUCs of 0.815 (95% CI: 0.763–0.868) and 0.803 (95% CI: 0.747–0.859), respectively. Youden’s index indicated that 37.5 HU and 38.5 HU were the best thresholds for the LV (sensitivity/specificity: 0.843/0.640) and DAo (sensitivity/specificity: 0.814/0.656), respectively. The negative predictive value and positive predictive value for the LV and DAo were 0.94/0.40 and 0.93/0.40, respectively. The AUCs of the IVS-LV, IVS-DAo and IVS were 0.651 (95% CI: 0.578–0.724), 0.622 (95% CI: 0.551–0.694), and 0.685 (95% CI: 0.622–0.747), respectively. DeLong test confirmed that there was no significant difference in AUC values between LV and DAo, and there was a significant statistical difference in AUC values of LV and DAo with IVS, IVS-LV, and IVS-DAo (*P* < 0.05, Table 7). The sensitivity and specificity of these indicators in diagnosing anemia under different thresholds were shown in Table 8. In the CDCT group, the LV and IVS-LV showed a favorable result in predicting anemia that produced AUCs of 0.831 (95% CI: 0.786–0.877) and 0.851 (95% CI: 0.808–0.894), respectively. The negative predictive value and positive predictive value for the LV and IVS-LV were 0.88/0.59 and 0.86/0.65, respectively. The AUCs of the DAo, IVS-DAo and IVS were 0.798 (95% CI: 0.748–0.847), 0.780 (95% CI: 0.729–0.831), and 0.562 (95% CI: 0.498–0.625), respectively. DeLong test confirmed that there was no significant difference in AUC values between IVS-LV and LV, but the difference in AUC values between IVS-LV and other indicators was statistically significant. There is no significant difference in AUC values of LV with DAo and IVS-DAo (Table 9). The optimal cutoffs for IVS-LV and LV were 9.5 HU (sensitivity/specificity: 0.767/0.778) and 40.5 HU (sensitivity/specificity: 0.817/0.697) (Table 10), respectively.

**TABLE 7:** DeLong test between AUC values of each index in LDCT group.

Indicators	Difference of AUC	95% CI	*P*
IVS ∼ LV	0.131	0.067–0.195	0.000*
IVS ∼ DAo	0.118	0.044–0.193	0.002*
IVS ∼ IVS-LV	0.033	–0.081–0.148	0.568
IVS ∼ IVS-DAo	0.062	–0.056–0.180	0.302
LV ∼ DAo	0.013	–0.046–0.071	0.677
LV ∼ IVS-LV	0.164	0.101–0.227	0.000*
LV ∼ IVS-DAo	0.193	0.106–0.280	0.000*
IVS-LV ∼ DAo	0.152	0.064–0.239	0.001*
IVS-LV ∼ IVS-DAo	0.029	–0.046–0.104	0.449
DAo ∼ IVS-DAo	0.181	0.122–0.239	0.000*

LDCT, low dose computed tomography; IVS, interventricular septum; LV, left ventricular cavity; IVS-LV, CT attenuation difference between LV and IVS; DAo, descending aorta; IVS-DAo, CT attenuation difference between DAo and IVS; AUC, area under the curve; 95% CI, 95% confidence interval. *The difference is significant between the two indicators.

**TABLE 8:** ROC analysis of LV and DAo in the LDCT group.

Threshold (HU)	LV			DAo		
		
	Sensitivity	Specificity	Youden’s index	Sensitivity	Specificity	Youden’s index
33.5	0.543	0.864	0.407	0.386	0.912	0.298
34.5	0.629	0.832	0.461	0.471	0.896	0.367
35.5	0.657	0.768	0.425	0.586	0.856	0.442
36.5	0.729	0.696	0.425	0.643	0.800	0.443
37.5	0.843	0.640	0.483	0.700	0.744	0.444
38.5	0.886	0.560	0.446	0.814	0.656	0.470
39.5	0.900	0.508	0.408	0.843	0.624	0.467
40.5	0.986	0.320	0.306	0.886	0.452	0.338
41.5	1	0.260	0.260	0.929	0.384	0.313

LDCT, low dose computed tomography; LV, left ventricular cavity; DAo, descending aorta; HU, Hounsfield Unit; ROC, receiver operating characteristic.

**TABLE 9:** DeLong test between AUC values of each index in CDCT group.

Indicators	Difference of AUC	95% CI	*P*
IVS ∼ LV	0.270	0.213–0.326	0.000*
IVS ∼ DAo	0.236	0.171–0.301	0.000*
IVS ∼ IVS-LV	0.289	0.212–0.367	0.000*
IVS ∼ IVS-DAo	0.218	0.132–0.304	0.000*
LV ∼ DAo	0.034	–0.009–0.076	0.122
LV ∼ IVS-LV	0.020	–0.010–0.050	0.194
LV ∼ IVS-DAo	0.052	–0.004–0.107	0.068
IVS-LV ∼ DAo	0.054	0.005–0.102	0.032*
IVS-LV ∼ IVS-DAo	0.071	0.024–0.119	0.003*
DAo ∼ IVS-DAo	0.018	–0.012–0.048	0.243

CDCT, conventional dose computed tomography; IVS, interventricular septum; LV, left ventricular cavity; IVS-LV, CT attenuation difference between LV and IVS; DAo, descending aorta; IVS-DAo, CT attenuation difference between DAo and IVS; AUC, area under the curve; 95% CI, 95% confidence interval. *The difference is significant between the two indicators.

**TABLE 10:** ROC analysis of LV and IVS-LV in the CDCT group.

Threshold (HU)	LV			IVS-LV		
		
	Sensitivity	Specificity	Youden’s index	Sensitivity	Specificity	Youden’s index
36.5/5.5	0.517	0.919	0.436	0.408	0.964	0.372
37.5/6.5	0.608	0.869	0.477	0.492	0.941	0.433
38.5/7.5	0.675	0.819	0.494	0.608	0.887	0.495
39.5/8.5	0.725	0.742	0.467	0.675	0.819	0.494
40.5/9.5	0.817	0.697	0.514	0.767	0.778	0.545
41.5/10.5	0.850	0.584	0.434	0.842	0.674	0.516
42.5/11.5	0.908	0.489	0.397	0.925	0.575	0.500
43.5/12.5	0.958	0.380	0.338	0.950	0.430	0.380
44.5/13.5	0.967	0.294	0.261	0.967	0.299	0.266

CDCT, conventional dose computed tomography; LV, left ventricular cavity; IVS-LV, CT attenuation difference between LV and IVS; HU, Hounsfield Unit; ROC, receiver operating characteristic.

## Discussion

Low-dose computed tomography (LDCT) is widely used in lung cancer screening and pulmonary disease diagnosis because of its effectiveness, ease of performance, and low radiation. To maximize the benefits of screening and enrich the clinical information, our study evaluated whether LDCT used for lung screening might help approximately estimate Hb levels and detect anemia. We compared the predicting performance of LDCT and CDCT on anemia from 5 indicators including the left ventricular (LV) cavity, descending aorta (DAo), interventricular septum (IVS), differences between the LV and IVS (IVS-LV), and differences between DAo and IVS (IVS-DAo). We found that IVS-LV and the LV from CDCT showed a favorable result in distinguishing between healthy people and patients with anemia. However, the LV and DAo from LDCT had satisfactory performance in detecting anemia. LDCT showed a comparable predictive effect with CDCT.

CT is one of the most commonly used clinical diagnostic imaging techniques because of its advantages of high temporal and spatial resolution, valuable 3-dimensional (3D) tomography information, and cost-effectiveness. Wójtowicz et al. (26) demonstrated that the smallest discernible difference between the ventricular wall and ventricular cavity was about 6–8 HU after analyzing CT images of 21 cases with severe anemia. Lan et al. (25) confirmed that IVS-LV gave the best results for diagnosing anemia with an AUC of 0.801. The CT attenuation difference of > 10 HU in men or > 12 HU in women would be an almost definite sign of severe anemia. Zhou et al. (27) considered the threshold of IVS-LV at 13.5 HU could obtain high diagnostic performance for severe anemia with the AUCs of 0.908 for men and 0.923 for women, respectively. Zopfs et al. (29) demonstrated that an approximation of serum Hb level and anemia can be conducted based on measurements of the LV and DAo in reconstructive virtual non-contrast (VNC) images. Cutoff values of mild, moderate, and severe anemia in VNC images are 39.2 HU (AUC = 0.857) and 33.6 HU (AUC = 0.879) for men and 37.6 HU (AUC = 0.833) and 32.7 HU (AUC = 0.932) for women, respectively. In our study, IVS-LV and the LV from CDCT produced AUCs of 0.851 (95% CI: 0.808–0.894) and 0.831 (95% CI: 0.786–0.877) in predicting anemia, respectively. The optimal cutoffs were 9.5 HU for IVS-LV and 40.5 HU for the LV cavity, respectively. This is consistent with previous research. Although CDCT images have shown certain value in the approximate evaluation of patients’ Hb levels, the practical application has great limitations due to high radiation levels.

After comparing predicting performance of LDCT and CDCT, we found that the predictive efficiency of LV and DAo in LDCT was superior to other indicators, with AUCs of 0.815 (95% CI: 0.763–0.868) and 0.803 (95% CI: 0.747–0.859), respectively. The optimal cutoffs were 37.5 HU for the LV and 38.5 HU for DAo, respectively. The AUC of DAo in LDCT was higher than that in CDCT. The possible reason might be that DAo was less affected by the adjacent spine in LDCT than in CDCT. The low correlation between IVS-LV and Hb level in LDCT may be explained in part by the fact that IVS was difficult to be distinguished clearly from the LV. Although a higher fluctuation of CT value in LDCT images, LDCT had satisfactory performance in detecting anemia.

To our knowledge, this study is the first to evaluate the diagnostic potential of thoracic LDCT scan in predicting anemia, which may enrich the application of chest LDCT screening. Generally, patients admitted to the hospital will have both a blood routine examination and CDCT scanning, but according to the screening protocol, subjects will not have the blood routine examination before receiving LDCT scanning. This study hopes to provide as much information as possible for the screening subjects and to prompt the subjects who may have anemia to perform blood routine examinations. We confirmed that the LV and DAo may contribute to clinical diagnosis by comparing the predicting performance of each indicator in LDCT and CDCT. The formula derived from linear regression analysis may provide an automated estimation of Hb level with every LDCT scan, which provided more diagnostic information for clinicians. When anemia is indicated by LDCT screening, patients can be directed to a blood examination. LDCT screening was a key component of the screening programs for early detection and early treatment, we will further investigate the additional value of LDCT screening to obtain more diagnostic information, such as the measurement of coronary artery calcium score and bone mineral density. We hope that our research can expand the application scope of LDCT screening to a certain extent, and improve the cost-effectiveness of LDCT screening.

Several limitations should not be ignored in this retrospective study. First of all, a relatively long interval between the CT scan and peripheral blood examination may be detrimental to the determination of the status of anemia. Second, the sample size of positive cases was small, especially the male anemic patients. Therefore, a large dataset was required for further studies. Third, the CDCT data and LDCT data were obtained from different groups of patients.

## Conclusion

Low dose computed tomography screening has a satisfactory performance in detecting anemia. The LV and DAo in LDCT can achieve high prediction performance for anemia, which is different from the best indicators in CDCT. Benefiting from low radiation, non-invasion, and convenience, LDCT-based estimation of Hb level is promising for clinical application and expanding the value of LDCT.

## Data availability statement

The original contributions presented in this study are included in the article/supplementary material, further inquiries can be directed to the corresponding author/s.

## Ethics statement

The Medical Ethics Committee of Tianjin Medical University Cancer Hospital approved this retrospective study (No. Bc2018039) with a waiver of informed consent.

## Author contributions

ZY and JZ: study design. JZ and JH: data collection. JZ and MW: data analysis. ZY and SL: supervision. JZ: manuscript writing. ZY and MW: revision. All authors contributed to the article and approved the submitted version.
